# The Impact of the Dermal Matrix in Tissue Reconstruction: A Bibliometric Perspective in Plastic Surgery

**DOI:** 10.3390/jfb15070189

**Published:** 2024-07-09

**Authors:** Daniel Pit, Bogdan Hoinoiu, Razvan Bardan, Teodora Hoinoiu

**Affiliations:** 1 Doctoral School, “Victor Babes” University of Medicine and Pharmacy Timisoara, E. Murgu Square, No. 2, 300041 Timisoara, Romania; daniel.pit@umft.ro; 2 Center for Advanced Research in Cardiovascular Pathology and Hemostaseology, “Victor Babes” University of Medicine and Pharmacy Timisoara, 300041 Timisoara, Romania; tstoichitoiu@umft.ro; 3 Department of Oral Rehabilitation and Dental Emergencies, Faculty of Dentistry, “Victor Babes” University of Medicine and Pharmacy Timisoara, P-ta Eftimie Murgu 2, 300041 Timisoara, Romania; 4 Interdisciplinary Research Center for Dental Medical Research, Lasers and Innovative Technologies, 300070 Timisoara, Romania; 5 Department XV, Discipline of Urology, “Victor Babes” University of Medicine and Pharmacy Timisoara, E. Murgu Square, No. 2, 300041 Timisoara, Romania; razvan.bardan@umft.ro; 6 Department of Clinical Practical Skills, “Victor Babes” University of Medicine and Pharmacy Timisoara, Eftimie Murgu Sq. Nr. 2, 300041 Timisoara, Romania

**Keywords:** dermal matrix, reconstruction of soft tissue defects, plastic surgery, bibliometrics

## Abstract

In the vast field of medical scientific research, few topics have managed to attract as much attention and mobilise academic resources as the use of dermal matrices in the reconstruction of soft tissue defects. In this study, we used bibliographic metrics such as co-authorship, keyword co-occurrence, and citations per document to analyse the relationship between the use of dermal matrices to reconstruct soft tissue defects caused by burns, tumours, and trauma. In addition, keyword analysis has highlighted the crucial role of technology in recent studies and the innovation brought about by the use of dermal matrices in the reconstruction of soft tissue defects. Keywords used in recent studies have revealed the critical role of technology in the development of the field. We extracted a set of 1329 research papers from the Web of Science Core Collection database between 2010 and 2024 that met our criteria. Through keyword analysis, we identified technology as a significant factor in recent studies. Our results showed that there is very little collaboration between authors on the topic and that most of them are from Asia. A significant number of articles on this topic come from the USA, China, Japan, Germany, the UK, and France. We discovered the top ten most cited sources analysing the use of dermal matrices in the reconstruction of soft tissue defects. Finally, we think that this study will be beneficial for our further research.

## 1. Introduction

Reconstruction of soft tissue defects, a key area of plastic and reconstructive surgery, represents a major challenge for clinicians and has a significant impact on patients’ quality of life. In an attempt to refine surgical techniques and provide optimal patient outcomes, the use of dermal matrices has emerged as an innovative and effective approach [1]. In this context, the use of dermal matrices has become increasingly common and important, representing an innovative option in the reconstruction of skin integrity and other soft tissue defects resulting from trauma, tumours, burns, and complex surgery [2,3,4]. Recent scientific studies have shown that dermal matrices function as supportive structural support, consisting mainly of collagen and other extracellular substances for tissue regeneration, and provide an effective solution for the reconstruction of soft tissue defects [5,6]. These characteristics make them ideal for use in soft tissue reconstruction and promote healing with both superior functional and aesthetic results [6,7,8]. Investigating the use of dermal matrices in the reconstruction of soft tissue defects will not only highlight the significant contributions of this technology to improving clinical practice. Still, it will also highlight the continued need for research and innovation in this vital area of reconstructive medicine [9,10,11].

However, before exploring the full potential of this approach, it is essential to understand the context in which the research is taking place and to identify emerging trends [12]. The considerable number of articles available illustrates the growing importance of this area of research. Advanced techniques, such as tissue engineering and bioprinting, have revolutionised how dermal matrices can be applied in reconstructive surgery [1,12,13].

This study aims to investigate in depth the use of dermal matrices in soft tissue defect reconstruction, exploring current trends, major contributions, and future research directions in this field. To do this, we will apply a range of analytical methods, including bibliometric and co-authorship analysis, to gain a comprehensive understanding of the current state of research and key issues influencing the evolution of this field [1,14]. Bibliometric analysis, using data analysis tools, allowed us to assess the number of articles available on this topic and to examine their characteristics, including keywords used, collaborative networks between authors and organisations, and the level of citation and impact of the research [14,15].

By exploring this topic, we aim to contribute to the consolidation of existing knowledge and find new opportunities for improving clinical practice in soft tissue defect reconstruction, with a focus on the effective and innovative use of dermal matrices.

This bibliometric study was undertaken to provide a comprehensive analysis of the research trends within the field of dermal matrices. Our objective was to gain a deeper understanding of the evolving research landscape, with a specific focus on identifying prevalent trends, fostering international collaboration, and ascertaining key knowledge gaps.

## 2. Materials and Methods

The evaluation and review of the scientific literature can be challenging due to the large volume of documents. For a comprehensive analysis of the literature from the perspective of bibliographic metrics, bibliometric analysis meets all criteria of rigor to create an objective overview of the level of knowledge on a particular subject [15]. Moreover, through bibliometric analysis, research trends can be identified or the geographical coverage of research on a particular subject can be assessed. Bibliometric analysis addresses two key aspects of research, namely performance analysis and scientific mapping [16]. Performance analysis is conducted using metrics such as the annual number of documents and citations or the impact of a document through the number of citations.

**Figure 1:**
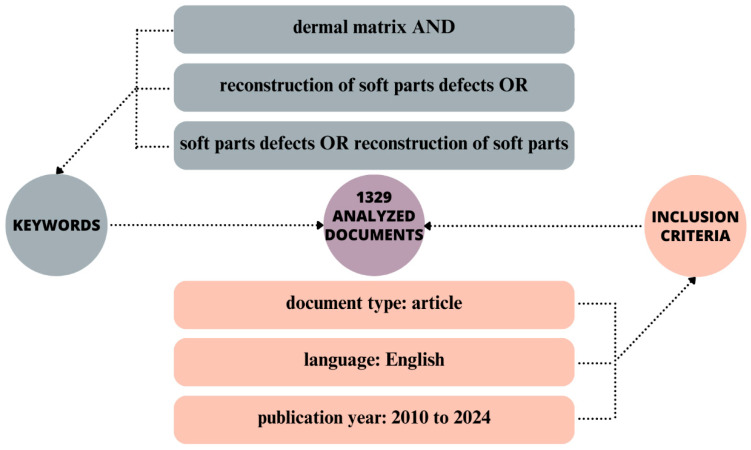
Data selection method.

The proposed analysis is based on data extracted from the Web of Science Core Collection database. In selecting relevant documents, we considered the relationship between the use of dermal matrices as a solution for the reconstruction of soft tissue defects caused by burns, tumours, or trauma. The keywords used in the search were “dermal matrix” AND “reconstruction of soft parts defects” OR “soft parts defects” OR “reconstruction of soft parts” The final number of 1329 documents included in the analysis was the result of applying language filters, including only documents written in English, and document type filters, including only articles published between 2010 and 2024. Graphically, the selection was made according to the scheme in Figure 1. Analysis of the obtained results was carried out using the software VOSviewer. The version we used in our work is 1.6.18.

The criteria for including documents in the analysis are the type of document, the language in which the document was written, and the year of publication of the document. The language criterion is generated by VOSviewer software’s ability to analyse documents written exclusively in English. The inclusion criterion regarding the year of publication is set for the period 2010–2024, so that the bibliometric analysis is performed on the newest documents on the subject of research. The selection criterion related to the document type is generated by the characteristics of the selected document type. Article-type documents are considered new and original and can be cited in other papers. Taking into account the fact that Web of Science frames a document in several categories (for example, Proceeding Paper or Review), we selected only article-type documents to eliminate the possibility of having duplicate documents in the data set [17]. Thus, the language exclusion criterion eliminated documents written in any language other than English, the publication year criterion excluded documents that were not published in the 2010–2024 period, and the document-type criterion excluded proceeding paper documents, review articles, book chapter, early access, educational material, and data paper. The application of these criteria allowed us to obtain a set of documents that corresponds both to the requirements imposed by VOSviewer software and to the requirements regarding the research objective of the work.

VOSviewer software was developed by Nees Jan van Eck and Ludo Waltman and can be used in various fields of research, such as economic [18] or medical. Over time, it has been improved through versions. VOSviewer creates maps using the distance approach method that highlights the relationships between the analysed items. The items can be authors, organisations, countries, keywords, documents, sources, cited references, cited sources, or cited authors. For these items, the links of co-authorship, co-occurrence, citation, bibliographic coupling, or co-citation through maps can be analysed. Each map can analyse only one type of item and one type of connection. The elements of a map are the items and the links between them. Some works in the literature refer to the item as a node in the network. Thus, the position of an item on the map determines the importance of the item in the analysis carried out. The closer it is to the centre of the image, the more important it is. Only one link can be established between two items. However, an item can have connections with every analysed item, which means that its importance in the analysis is very high. The size of an item on the map indicates its relevance defined by the number of appearances, citations, or documents, depending on the type of analysis. The distance between two items shows the strength of the relationship between them. The smaller the distance, the stronger the relationship and vice versa. Items that have several links in common can be associated with a cluster identified within the map with a distinct colour. The stronger links the items in a cluster have, the better the cluster they belong to is defined within the network. In carrying out each type of analysis of the relationships between the items, inclusion criteria established by minimum thresholds for the items to be analysed can be used (e.g., the number of citations of a document or the number of occurrences of a keyword) [17].

The main types of analysis targeted scientific co-authorship, keyword co-occurrence, and citations per document. Each type of analysis used a specific unit of measurement, including authors, documents, sources, institutions, or countries [18,19].

## 3. Results and Discussion

### 3.1. Keyword Analysis

The co-occurrence analysis of keywords aims to form a theoretical framework regarding the research trends associated with the use of dermal matrices in tissue reconstruction. This fact can be achieved by identifying the most used keywords within the articles in the data set. Moreover, the analysis of keywords according to the year of appearance traces the evolution of the subject throughout the analysed period.

**Figure 2:**
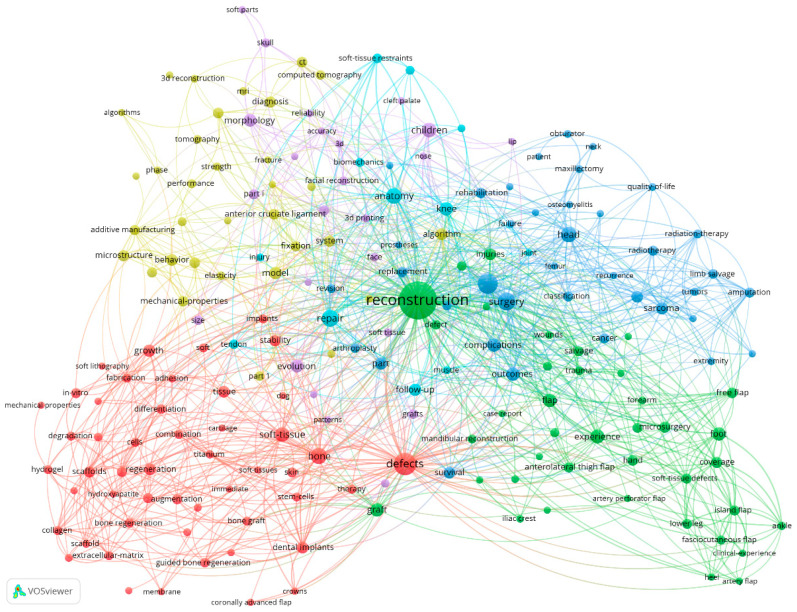
Keyword co-occurrence analysis by research themes, processed with VOSviewer.

Keyword analysis builds a graphical network of keywords that have the highest occurrence in the research topic concerning the use of dermal matrices in the reconstruction of soft tissue defects. The results of the analysis can identify two essential aspects of researching this topic: the associated research trend and relevant themes. The software identified 7710 keywords across the 1329 documents, of which 196 meet the minimum threshold of seven occurrences together. The keyword network representing the research themes associated with our subject of analysis is shown in Figure 2. The 196 keywords form six clusters, highlighted by different colours, each outlining a sub-theme associated with the analysed topic. In terms of frequency of occurrence, “reconstruction” stands out with a total link strength of 584 and occurrence of 238, followed by “defects”, “management”, “surgery”, and “repair”, each having a total link strength of 241, 168, 157, and 133 and occurrence of 79, 61, 50, and 42, respectively. However, in the graphical sample of keywords represented by applying the filter for a minimum of seven occurrences of keywords, we observed that the search term “dermal matrix” was not included.

The size of the nodes in the network highlights the importance of the keywords. The larger the node and the more connections it has, the greater the central interest in the researched topic. Additionally, the distance between each pair of nodes reflects the frequency of their association in the same document. The proximity of words to the centre of the image forms another area of interest that includes terms such as “reconstruction”, “replacement”, “repair”, “surgery”, “complications”, “defect”, “soft tissue”, and “arthroplasty”. Following the keyword analysis, we can affirm that our research topic on the use of dermal matrices in the reconstruction of soft tissue defects is quite novel, a fact corroborated by the limited occurrence of the term “dermal matrix” within the analysis.

The keyword “dermal matrix” is not included in the figure, because it does not meet the minimum number of seven occurrences set for the construction of the map. This minimum threshold was set to 7 to avoid overlapping items and to obtain an easily interpretable bibliographic network. However, even with this number of occurrences, we find the associated keywords “extracellular matrix” and “extracellular-matrix” with 24 links and 9 occurrences and, respectively, 28 links and 12 occurrences. If we had set the number of occurrences to the minimum threshold of 5, then the associated keywords “acellular dermal matrix” with 12 links and 5 occurrences and “matrix” with 26 links and 6 occurrences would have been displayed, and “extracellular matrix” would have registered 29 connections and “extracellular-matrix” would have registered 33 connections. These results show the most important characteristic of the analysed subject, namely the novelty of the subject. If we had analysed this last result through overlay visualisation, we would have noticed that the years of appearance of these terms would have been after 2016, which confirms the novelty of the subject. Considering the fact that the research topic is part of the medical sphere, we must take into account that the studies extend over a longer period of time.

**Figure 3:**
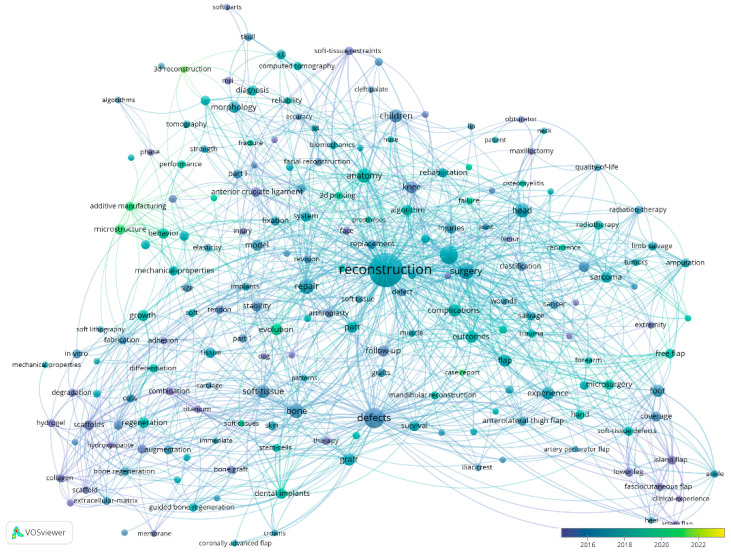
Keyword co-occurrence analysis by years processed with VOSviewer.

Figure 3 illustrates the network of keyword connections, showing the usage of keywords over time through different colours. Dark blue shades indicate the use of keywords in research published in 2015, while lighter blue shades denote keywords used in more recent research. Nodes marked in green and yellow are associated with more recent research, specifically those published after 2020 and up to 2024. In studies from 2015, keywords such as “hydrogel”, “defects”, “collagen”, and “biomechanics” were more prevalent, whereas, from 2020 onwards, terms like “3D reconstruction”, “evolution”, “soft tissues”, “microstructure”, and “additive manufacturing” have become more prominent.

Figure 3 outlines the research trend for the use of dermal matrices in the reconstruction of soft tissue defects, revealing a growing interest in the involvement of technology such as 3D reconstruction and 3D printing, as well as the identification of new treatment methods like microstructure, optimisation, additive manufacturing, and evolution. Overall, the keyword analysis has allowed us to identify technology as an influential factor in recent studies, alongside the novelty of the approach to using dermal matrices in the reconstruction of soft tissue defects.

### 3.2. Co-Authorship Analysis among Authors

The research interest in the medical field concerning the use of dermal matrices in the reconstruction of soft tissue defects can be assessed by the number of authors who tackle this topic but, more specifically, by the co-authorship relationships established through the progression of scientific research. The analysis in this section aims to identify the most robust co-authorship relationships on the analysed subject, thereby visualising the most relevant research collectives.

**Figure 4:**
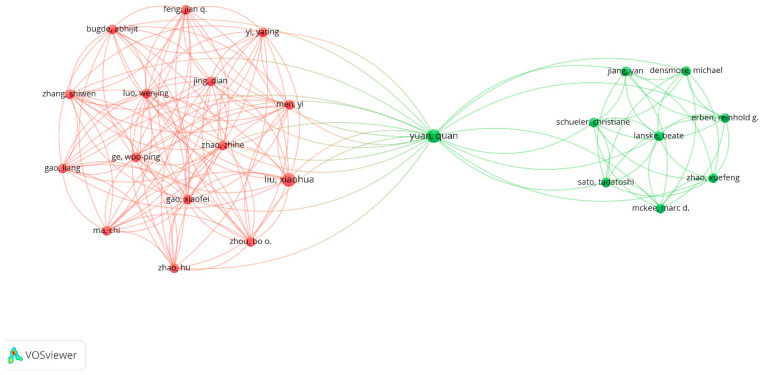
The scientific co-authorship network between authors was processed with VOSviewer.

Out of a total of 6668 authors identified in the analysed articles, 479 of them have been cited at least 50 times. However, the level of collaboration between them is very low, making a graphical representation of all 479 authors less meaningful. On the other hand, a group of 24 authors managed to form research teams that hold significant importance for the analysed topic. Figure 4 distinguishes the clusters formed by the authors who collaborated on this subject.

As can be seen, distinguished by colours, there are only two well-defined clusters, with author Yuan Quan acting as the connecting link between them. The red cluster comprises 15 authors, while the green cluster consists of 9 authors. The article “Increased Osteopontin Contributes to Inhibition of Bone Mineralization in FGF23-Deficient Mice” elaborated by Quan Yuan et al. in 2014 [20] combines numerous empirical analysis methods such as biochemical analyses, bone histology, mCT analyses, histomorphometry, immunohistochemistry, quantitative real-time PCR, electron microscopy, and immunolabeling or statistics to identify the reason why mineralisation inhibitors accumulate in the extracellular matrix of bones that generate soft tissue problems. Although the study did not refer to the dermal matrix, good practices, the combination of methods of analysis, and evaluation of the obtained results must be taken into account in order to carry out future empirical studies aimed at the use of dermal matrices in the treatment of soft tissue problems. Moreover, the group of authors was affiliated with academic or medical organisations in Boston, USA, Chengdu, China, Beijing, China, Vienna, Austria, and Montreal, Canada. Thus, this study also confirms good practices in terms of scientific collaboration relationships.

The article “Tissue clearing of both hard and soft tissue organs with the PEGASOS method” developed by the authors from the red cluster, together with Quan Yuan [21], empirically approached the PEGASOS method to obtain new information in body imaging. This article mainly focused on the part of tissues, both soft and hard, but the research methods and the results obtained confirmed the fact that the co-authorship led the authors to innovative and relevant discoveries for future studies. Even if the dermal matrix part was not directly addressed in this study, the innovation elements related to soft tissues can contribute to the development of our subject, namely the use of dermal matrices in the reconstruction of soft tissue tissues. In addition, in order for the results obtained on our subject of analysis to be validated and correct, it is necessary to have a very well-documented theoretical basis, the previously mentioned study being part of it.

### 3.3. Co-Authorship Analysis among Organisations

The co-authorship analysis between the organisations research collaboration in a specific area of scientific interest regarding the use of dermal matrices as a solution for the reconstruction of soft tissue defects. This approach allows us to analyse the behaviour of research teams and their network of relationships, identifying the most influential organisations in the field of research.

**Figure 5:**
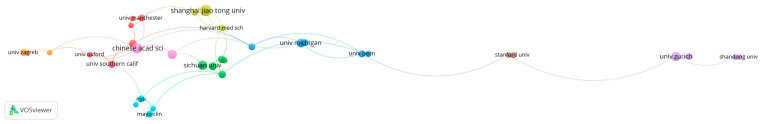
The scientific co-authorship network between organisations processed with VOSviewer.

The software VOSviewer identified a total of 2114 organisations engaged in research related to the use of dermal matrices in the reconstruction of soft tissue defects. Of these, 53 organisations were found to be relevant due to a high number of citations (over 50) and a large number of published documents (at least five). Graphically, Figure 5 presents only 35 of the 53 organisations that have made efforts in researching the subject and advancing the level of knowledge thanks to the collaborative relationships that have developed among them.

The red cluster comprises six universities from the United States, the United Kingdom, and Ireland, including University College London, University of Bristol, University College Dublin, University of Manchester, University of Oxford, and the University of Southern California. The green cluster consists of five organisations, including two hospitals (Hospital for Special Surgery and Massachusetts General Hospital) and three universities (Harvard, Ohio, and Sichuan). In the blue cluster, five other universities stand out: Medical University of Vienna, Queen Mary University of London, Radboud University Nijmegen, University of Bern, and University of Michigan. The University of Michigan leads in terms of the number of documents, having published 12 articles. The yellow, turquoise, and violet clusters each comprise four organisations. Notably, in the yellow cluster, Shanghai Jiao Tong University emerges as the most scientifically productive organisation, publishing 21 articles on the studied topic. In the turquoise cluster, the Mayo Clinic is present, while the violet cluster is distinguished by its distance from the centre of the image, indicating a limited research relationship with other institutions of interest on the topic.

The orange cluster with three components and the pink cluster with two components complete the central image but are characterised by limited connections with other organisations. The last cluster, the brown cluster, consists of two universities (Goethe University Frankfurt and Stanford University) and has a low link strength. The results of this analysis indicate that the research topic involving the use of dermal matrices in the reconstruction of soft tissue defects is primarily approached by academia, with most institutions being universities, while the presence of private medical organisations is nearly non-existent. Furthermore, the network formed through research collaborations reveals an intercontinental coverage of the area of interest in this subject.

The co-authorship relations between the organisations additionally validate the quality of the results of the studies carried out through the international recognition of the good practices they have promoted in the scientific environment. In addition, the identified collaborations between universities and medical research centres provide the authors with an extensive material basis for carrying out studies but, at the same time, ensure the transfer of information to increase the quality of the results regarding the applications of dermal matrices in tissue reconstruction.

### 3.4. Co-Authorship Analysis among Countries

The bibliometric analysis of scientific co-authorship between countries aims to identify which are the most productive countries on the research subject and which bring the greatest contributions in terms of the characteristics of innovations specific to our subject.

**Figure 6:**
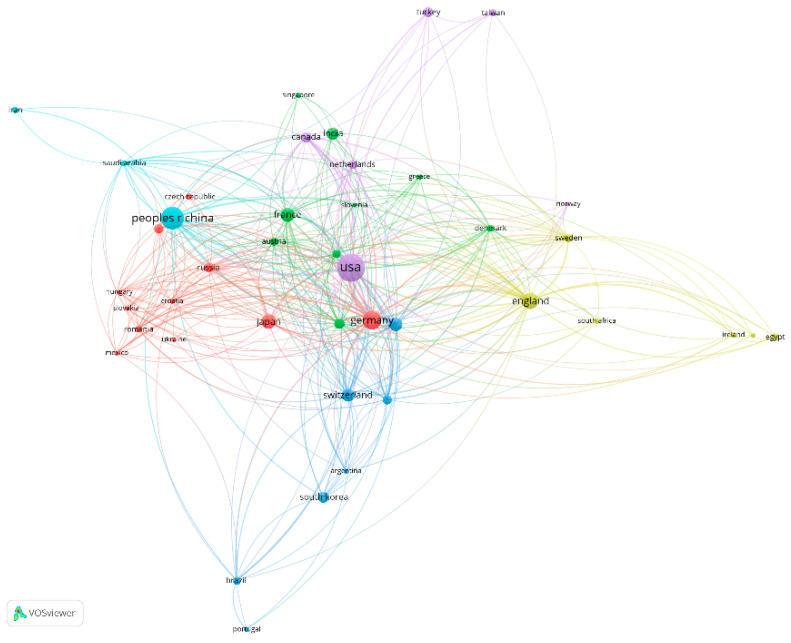
The scientific co-authorship network between countries processed with VOSviewer.

By using countries as a unit of analysis for scientific co-authorship, the graphical representation in Figure 6 was obtained. Out of a total of 81 countries involved in the study, only 42 met the minimum inclusion criteria of at least five documents published and a minimum of 50 citations. These thresholds allow us to identify the most relevant countries by validating the obtained results within the scientific community and the most productive countries through a high number of publications.

Although, in the earlier analysis of co-authorship among authors, many authors with names of Asian origin were notable, the results of this analysis place the United States in the lead for the number of documents and citations, with a significant gap until China, Germany, the United Kingdom, France, and Japan. Nevertheless, all the aforementioned countries have managed to establish international recognition by validating their research efforts through the publication of numerous articles and the acquisition of citations.

As can be seen, the red cluster is the largest, comprising 11 countries, with Germany and Japan showing the most significant metrics. In the green cluster, of the nine components, France records a total of 2021 citations and 76 articles, making it the top country within the cluster. Unlike the red cluster, the green cluster occupies a larger space in the network, with the distance between nodes indicating slightly weaker collaboration.

The blue cluster contains seven elements and is characterised by countries with less recognition in medical research, such as Argentina, Brazil, South Korea, and Portugal. However, Italy and Switzerland are at the top regarding the validation of results on the analysed subject. The yellow cluster, made up of seven countries, is situated far to the left of the figure, forming a collaboration network characterised by a low number of connections and weak link intensity. The fifth cluster, violet, contains the most significant country for our research subject from a publication standpoint: namely, the United States. Nevertheless, the internal links within the cluster are not very strong. Finally, the turquoise cluster is limited to three elements, with China making the most significant contributions regarding the analysed topic.

In conclusion, the co-authorship analysis among countries forms an internationalised research framework, in which countries such as the United States, China, Germany, the United Kingdom, France, and Japan are leaders in research. This is validated by the recognition of the results through a considerable number of citations and articles published on our research topic. It is important to note that none of the clusters are isolated from the others, with their overlap indicating a complex and diverse scientific partnership without geographical or other specific limitations.

### 3.5. Document Citation Analysis

**Figure 7:**
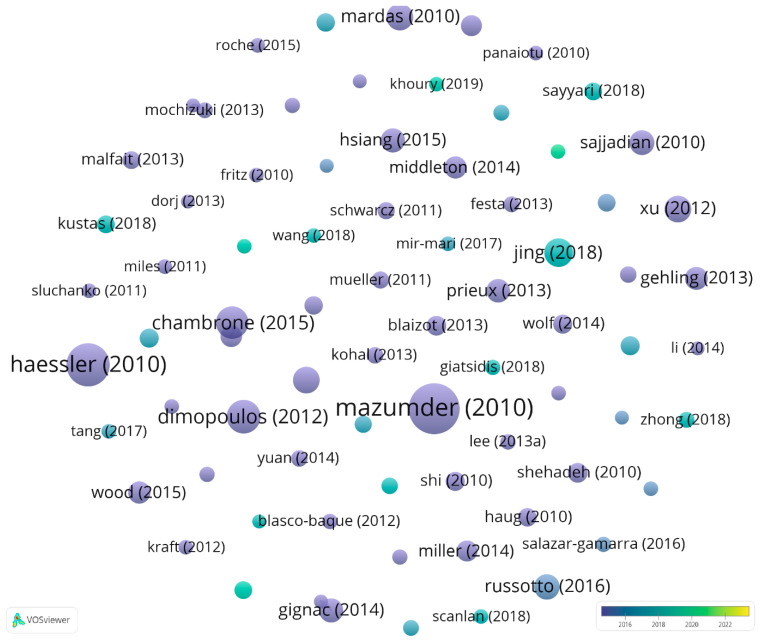
The network of citations on documents processed with VOSviewer.

Document citation analysis focuses on identifying the most important documents that study the use of dermal matrices in the reconstruction of soft tissue defects, using the number of citations as a benchmark for confirming the quality, centrality, and relevance of the articles used in the analysis. From the 1329 articles selected, we considered only the 76 documents that surpassed the minimum threshold of 50 citations as significant. The graphical representation of the network formed from the articles included in the analysis is shown in Figure 7.

Because the software did not identify shared citations among the selected articles in other scientific documents, we used the time–axis overlay representation to capture the publication years for the most important articles on the analysed subject. As observed, the article published by Mazumder (2010) recorded the highest number of citations, 744, followed by Haessler (2010) with 527 citations and Dimopoulos (2012) with 311. While Mazumder, Hastie, and Tibshirani (2010) developed a calculation algorithm to generate solutions and resolve problems in various fields, Haessler et al. (2010) focused their research efforts on performing tomographic reconstruction that combines attosecond and angstrom resolutions to provide a dynamic perspective of intramolecular imaging. Also, from the imaging spectrum, Dimopoulos et al. (2012) explored in their article the role of MRI imaging in a set of three recommendations targeting the implementation of brachytherapy for cervical cancer based on 3D images. The next document in terms of citations was published in 2015 and belongs to Chambrone and Tatakis. The authors conducted a very relevant study for our topic. Specifically, they developed an article that bridges the theoretical and practical aspects of medicine, conducting a study that outlines the most efficient treatment methods for covering soft tissue root defects of the recession type in daily clinical practice. The results showed that procedures involving the use of subepithelial connective tissue, coronally advanced flaps plus acellular dermal matrix grafts, enamel matrix derivative, or the collagen matrix achieved clinical improvements.

In formulating a conclusion regarding the analysis of citations per document, we can mention that, through the generated map, we identified the relevant documents for researching the relationship between dermal matrices and the reconstruction of soft tissue defects. Moreover, we observed a higher intensity of citations for articles published at the beginning of the analysis period compared to more recent articles. This is due to the specificity of the medical field, which involves using clinical studies to obtain relevant results, and the difficulty in reproducing some of them, as many newer articles are based on previous clinical studies that have been improved upon.

### 3.6. Analysis of Citations per Sources

**Figure 8:**
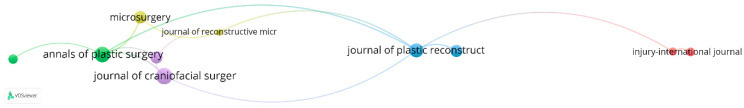
The network of citations on sources processed with VOSviewer.

The analysis of citations per source aims to identify the most reliable publications that contain studies on the use of dermal matrices in the reconstruction of soft tissue defects. Among the 1329 articles in our data, a total of 696 sources were identified, of which 29 had more than five documents that addressed the analysed topic and were cited at least 50 times. Figure 8 illustrates only those sources that form a shared citation network, as another representation of the sources would be irrelevant.

Based on the number of citations, Table 1 presents the top 10 most important publications that examine the use of dermal matrices in the reconstruction of soft tissue defects, using the number of citations as the method of analysis. As displayed in the table, all the publications have a medical focus and demonstrate reliability through both the high number of citations and the large number of documents relevant to the studied topic.

The publications listed in Table 1 represent the most influential sources in the field, having been widely cited by peers and contributing significantly to the current body of knowledge on the topic. These sources provide key insights into the application of dermal matrices in reconstructing soft tissue defects and offer evidence-based data and approaches that are crucial for advancing clinical practice.

These highly regarded publications not only discuss the efficacy of dermal matrices in treating soft tissue defects but also explore various aspects of the procedure, including novel techniques, materials, and long-term outcomes. Their work serves as a cornerstone for ongoing research and clinical innovation in the field.

The prominence of these sources underscores their pivotal role in shaping the current understanding and future developments in the use of dermal matrices for soft tissue reconstruction. Researchers and practitioners alike rely on the findings presented in these publications to guide their work and inform their approaches to patient care.

In summary, Table 1 highlights the critical contributions of these top 10 sources to the ongoing dialogue and advancement in the use of dermal matrices for reconstructing soft tissue defects. Their work continues to influence and inspire further exploration in this vital area of medical research.

## 4. Conclusions

The results of this analysis show that *the Journal of Biomedical Materials Research Part A* and the *Journal of Biomedical Materials Research Part B—Applied Biomaterials* are the most influential publications found in the analysed data, obtaining 633 citations and 533 citations, respectively. Another trusted source recognised for studies on the research topic is Plastic and Reconstructive Surgery, which stands out with a total of 420 citations and 16 documents. Knee Surgery, Sports Traumatology, Arthroscopy, and Clinical Oral Implants Research are equally recognised academically in terms of citations (416) and have 11 and 10 articles on the studied topic, respectively.

By reviewing all the types of analysis performed and summarising the results, we conclude that the bibliometric analysis of the research topic concerning the use of dermal matrices in the reconstruction of soft tissue defects has helped us identify the considerable scope of the subject in the scientific literature through the large number of associated articles available. Moreover, keyword analysis allowed us to identify technology as an influential factor in recent studies, alongside the novelty of the approach to using dermal matrices in the reconstruction of soft tissue defects [19,22,23].

The co-authorship analysis among authors confirmed the novelty of the topic due to the low level of cooperation identified through the limited network of relationships and nodes. Co-authorship among organisations placed the research topic predominantly in the academic university environment and, together with co-authorship among countries, confirmed the high level of geographical spread of the research, with collaborative relationships being both continental and intercontinental [22,24,25].

The analysis of citations per document and source succeeded in identifying the most relevant articles and publications that can be explored for conducting specialised literature reviews or systematic reviews.

In conclusion, the analysis performed on the research topic on the use of dermal matrices in the reconstruction of soft tissue defects revealed a significant breadth in the literature, highlighting the technology as an influential factor and the novelty of the approach. Co-authorship between authors and organisations also confirmed the novelty of the topic and its predominantly academic placement, with a wide geographical spread and collaborations both continentally and intercontinentally. The citation analysis identified relevant articles and publications, providing a solid basis for future literature reviews or systematisations.

The results of the bibliometric analysis can be used both by researchers to identify possible collaborations that they can develop on the research topic or to identify the most suitable journals for their studies, as well as by political decision makers when they allocate resources in the field of medical research. The use of dermal matrices as a solution for soft tissue defects is a subject characterised by innovation, novelty, and a high research interest, which means that funds must be allocated to reach the maturity of the subject and to spread its applicability globally.

The field of soft tissue repair is undergoing rapid evolution, with significant advancements in biomaterials, stem cell therapy, 3D bioprinting, and gene therapy [26,27,28]. These innovative approaches hold great promise for improving patient outcomes and advancing the capabilities of regenerative medicine. Each area of research offers unique insights and potential applications, as demonstrated by recent studies and publications in the field. The incorporation of scaffolds such as dermal matrices into these strategies serves to enhance their effectiveness, providing structural support and bioactive signals that are necessary for successful tissue regeneration [29].

## Figures and Tables

**Table 1:** Top 10 Most cited sources analysing the use of dermal matrices in the reconstruction of soft tissue defects.

Source	Citations	Documents	Journal Impact Factor 2023	Category	Publication Frequency
Journal of Biomedical Materials Research Part a	633	26	3.9	Materials Science, Biomaterials, Engineering, Biomedical	12 issues/year
Journal of Biomedical Materials Research part b—applied biomaterials	533	28	3.2	Materials Science, Biomaterials, Engineering, Biomedical	8 issues/year
Plastic and Reconstructive Surgery	420	16	3.2	Surgery	12 issues/year
Knee Surgery Sports Traumatology Arthroscopy	416	11	3.3	Surgery, Sport Sciences, Orthopaedics	12 issues/year
Clinical Oral Implants Research	416	10	4.8	Dentistry, Oral Surgery & Medicine, Engineering, Biomedical	12 issues/year
Journal of Clinical Periodontology	276	5	5.8	Dentistry, Oral Surgery & Medicine	12 issues/year
Injury-International Journal of the Care of the Injured	273	8	2.2	Critical Care Medicine, Surgery, Emergency Medicine, Orthopaedics	12 issues/year
Annals of Plastic Surgery	272	27	1.4	Surgery	12 issues/year
Clinical Orthopaedics and Related Research	215	9	4.2	Surgery, Orthopaedics	12 issues/year
Medical Physics	205	12	3.2	Radiology, Nuclear Medicine & Medical Imaging	12 issues/year

## Data Availability

No new data were created or analyzed in this study. Data sharing is not applicable to this article.
